# Impact of advertisements promoting candy-like flavoured e-cigarettes on appeal of tobacco smoking among children: an experimental study

**DOI:** 10.1136/tobaccocontrol-2015-052593

**Published:** 2016-01-18

**Authors:** Milica Vasiljevic, Dragos C Petrescu, Theresa M Marteau

**Affiliations:** Behaviour and Health Research Unit, University of Cambridge, Cambridge, UK

**Keywords:** Electronic nicotine delivery devices, Advertising and Promotion, Media, Nicotine

## Abstract

**Background:**

There are concerns that the marketing of e-cigarettes may increase the appeal of tobacco smoking in children. We examined this concern by assessing the impact on appeal of tobacco smoking after exposure to advertisements for e-cigarettes with and without candy-like flavours, such as, bubble gum and milk chocolate.

**Methods:**

We assigned 598 English school children (aged 11–16 years) to 1 of 3 different conditions corresponding to the adverts to which they were exposed: adverts for flavoured e-cigarettes, adverts for non-flavoured e-cigarettes or a control condition in which no adverts were shown. The primary endpoint was appeal of tobacco smoking. Secondary endpoints were: appeal of using e-cigarettes, susceptibility to tobacco smoking, perceived harm of tobacco, appeal of e-cigarette adverts and interest in buying and trying e-cigarettes.

**Results:**

Tobacco smokers and e-cigarette users were excluded from analyses (final sample=471). Exposure to either set of adverts did not increase the appeal of tobacco smoking, the appeal of using e-cigarettes, or susceptibility to tobacco smoking. Also, it did not reduce the perceived harm of tobacco smoking, which was high. Flavoured e-cigarette adverts were, however, more appealing than adverts for non-flavoured e-cigarettes and elicited greater interest in buying and trying e-cigarettes.

**Conclusions:**

Exposure to adverts for e-cigarettes does not seem to increase the appeal of tobacco smoking in children. Flavoured, compared with non-flavoured, e-cigarette adverts did, however, elicit greater appeal and interest in buying and trying e-cigarettes. Further studies extending the current research are needed to elucidate the impact of flavoured and non-flavoured e-cigarette adverts.

## Introduction

Electronic cigarettes (e-cigarettes) have the potential for benefit if they assist tobacco smokers to quit or harm if they result in children smoking tobacco cigarettes.^1^ While uncertainty remains regarding their potential for benefit, evidence is accumulating to suggest that e-cigarettes can successfully be used as cessation aids by smokers.^2^ Uncertainty also remains regarding their potential for harm, with some cross-sectional studies reporting that e-cigarette use among children is associated with intention to smoke tobacco,^3^ ^4^ and a recent longitudinal study of US individuals aged 16–26 years finding that use of e-cigarettes at baseline is associated with progression to tobacco smoking 1 year later.^5^

E-cigarettes are now the most commonly consumed nicotine product among children in countries with strong tobacco control policies.^6^ ^7^ A recent WHO report suggested that e-cigarettes could provide a gateway to tobacco smoking by: (1) increased initiation of nicotine use which would not have occurred if e-cigarettes did not exist and/or (2) product swap from e-cigarettes to tobacco among those who initially become addicted to nicotine via e-cigarettes.^8^ The report also suggested that e-cigarettes could renormalise tobacco smoking by enhancing the attractiveness of tobacco smoking.^8^ On the basis of the social learning theory, users of cigarettes of any kind may serve as social role models to children observing them.^9–11^ Whereas the gateway hypothesis proposes that the impact of e-cigarettes on tobacco smoking is indirect, whereby children and adult non-smokers may be at risk of migrating to tobacco smoking after using e-cigarettes, the renormalisation hypothesis proposes a direct route, whereby the presence of e-cigarettes in the public arena is sufficient to encourage children and adult non-smokers to start smoking tobacco without the intervening step of first using e-cigarettes. In the present study our primary aim was to test the impact of exposure to e-cigarette adverts on the appeal of tobacco smoking among children (renormalisation). Our secondary aim was to test the impact of exposure to e-cigarette adverts on the appeal of using e-cigarettes (gateway hypothesis).

The increasing experimentation and use of e-cigarettes by children could be due, in part, to the lack of a regulatory framework surrounding e-cigarette advertising.^12^ ^13^ For example, in the USA, exposure of children to TV e-cigarette adverts increased by 256% from 2011 to 2013.^14^ Of particular interest are the possible attitudinal and behavioural shifts in children that may result from marketing of flavoured e-cigarettes. E-cigarettes are marketed in 7764 different flavours.^15^ Particularly concerning is the use of candy-like flavours (eg, bubble gum, milk chocolate) used successfully in the past to attract children to tobacco smoking.^16^ As described above, candy-like flavoured e-cigarettes may serve as a renormalising or gateway product to tobacco cigarettes. Even if candy-like flavoured e-cigarettes do not serve as gateway, or renormalising products into tobacco smoking, they may increase experimentation and eventual addiction to nicotine delivered through e-cigarettes, a substance with adverse consequences for the developing adolescent.^8^ ^17^ The above concerns are supported by research into candy-flavoured and liqueur-flavoured traditional tobacco products which were heavily marketed to young people since the 1970s until 2009 when regulations were imposed.^18^ ^19^ Internal tobacco industry documents reveal that the use of flavours in nicotine products targets potential new young customers: ‘It's a well known fact that teenagers like sweet products. Honey might be considered’ (memo recommending the company consider Coca-Cola or other sweet-flavoured cigarettes).^20^ Internal documents describing research by the tobacco industry also report that younger smokers are indeed more open to unique and exotic flavours^21^ with these products, also making smoking appear less risky and more acceptable.^16^ ^22^

While this evidence suggests the potential for e-cigarettes to increase the appeal of tobacco smoking, against this, the increasing rates of e-cigarette use among children has not been paralleled by an increase in rates of tobacco smoking. In the USA, e-cigarette use tripled from 2013 to 2014 among high schoolers rising from 4.5% to 13.4%, and among middle school students increasing from 1.1% to 3.9%, while tobacco smoking rates declined from 15.8% to 9.2%, and 4.3% to 2.5%, respectively, among these two groups from 2011 to 2014.^7^ These figures are mirrored in England where e-cigarette use has risen from 5% in 2013 to 8% in 2014 among individuals aged 11–18 years,^23^ while tobacco smoking has declined from 5% in 2010 to 3% in 2014 among children aged 11–15 years.^24^

To date, there is no empirical evidence concerning the impact of marketing of e-cigarettes with or without flavours on the appeal of tobacco smoking in children. This study aims to address this gap by estimating the impact of advertisements for candy-like flavoured and non-flavoured e-cigarettes on the appeal of tobacco smoking and e-cigarette use. The study was conducted among English school children in the age range 11–16 years when children are most likely to try and initiate daily tobacco smoking.^25^

## Methods

### Design

A between-subjects experiment with one independent factor of three levels corresponding to the advertisements to which participants were exposed:

- Advertisements of candy-like flavoured e-cigarettes
- Advertisements of non-flavoured e-cigarettes
- No advertisements (control condition)

### Participants

These comprised 598 English children aged 11–16 years attending two schools, one in Cambridgeshire and one in Hampshire (for demographics see table 1). Randomisation was successful; there were no significant differences between the three experimental groups on any of the characteristics.

**Table 1:** Participant demographic characteristics, e-cigarette use and tobacco smoking prevalence of full sample

	Control (n=201)	Flavoured ads (n=206)	Non-flavoured ads (n=191)	Total (n=598)
Age—M (SD)	13.13 (1.43)	13.10 (1.47)	13.27 (1.47)	13.16 (1.46)
Gender—female, % (n)	48.8 (98)	49.5 (102)	46.1 (88)	48.2 (288)
Ethnicity—white, % (n)	74.1 (149)	80.1 (165)	82.2 (157)	78.8 (471)
E-cigarette awareness—yes, % (n)	87.6 (176)	87.4 (180)	90.6 (173)	88.5 (529)
E-cigarette use—yes, % (n)	11.4 (23)	11.7 (24)	13.6 (26)	12.2 (73)
Cigarette use—yes, % (n)	9.5 (19)	7.8 (16)	10.5 (20)	9.2 (55)
Cigarette experimentation—yes, % (n)	14.9 (30)	9.2 (19)	14.1 (27)	12.7 (76)

Those who reported ever having smoked tobacco or e-cigarettes were excluded from the analyses (n=127) resulting in a final sample of 471 participants (for demographics of the final sample see table 2). This sample size provided over 90% power at α=0.05 to detect a medium-sized effect of either type of advert on the appeal of tobacco smoking (based on a study of the impact of standardised tobacco packaging using a similar endpoint).^26^

**Table 2:** Participant demographic characteristics of final sample

	Control (n=157)	Flavoured ads (n=166)	Non-flavoured ads (n=148)	Total (n=471)
Age—M (SD)	13.13 (1.43)	13.10 (1.47)	13.27 (1.47)	13.06 (1.48)
Gender—female, % (n)	45.9 (72)	51.2 (85)	47.3 (70)	48.2 (227)
Ethnicity—white, % (n)	75.2 (118)	80.1 (133)	81.1 (120)	78.8 (371)

### Intervention

Twenty-four adverts (12 for candy-like flavoured and 12 for non-flavoured e-cigarettes) were chosen and pilot tested from a larger sample of adverts sampled from the Stanford Adverts Repository.^27^ Two authors (MV and DCP) selected adverts that either explicitly showed candy-like flavours or not. With the exception of flavouring descriptions, care was taken to choose adverts as similar as possible in all other aspects (eg, showing an e-cigarette pack with an e-cigarette next to it), including the presence of a person (with three adverts in each of the e-cigarette advert conditions showing a person using an e-cigarette).

### Measures

#### Primary endpoint

##### Appeal of tobacco smoking

This was measured using 3 of the 11 bipolar items used by Ford *et al*^26^ to measure tobacco pack appeal. We asked participants: ‘Please cross the circles that best describe how you feel about smoking tobacco cigarettes’: *unattractive-attractive*, *not cool-cool* and *boring-fun* (rated from 1 to 5, with 1 denoting lowest appeal and 5 denoting highest appeal). Items were averaged into a single index (α=0.85).

#### Secondary endpoints

##### Appeal of using e-cigarettes

This was measured with an adapted version of the scale used to assess appeal of tobacco smoking, asking ‘Please cross the circles that best describe how you feel about using e-cigarettes’ (α=0.87).

##### Perceived harm of smoking tobacco cigarettes

This was measured using three items developed by Wakefield *et al*:^28^ ‘Smoking can harm your health’ rated from *1=Strongly disagree* to *5=Strongly agree*, ‘How dangerous do you think it is to smoke more than 10 cigarettes a day?’, and ‘How dangerous do you think it is to smoke one or two cigarettes occasionally?’ both rated on five-point scales, *1=Not very dangerous* to *5=Very dangerous*. The inter-item reliability was low for this scale (α=0.53). We therefore assessed this using the composite score and separately just the first item which has been most often used in the literature.

##### Susceptibility to tobacco smoking

Three items based on the scale of Pierce *et al*^29^ and adapted by Ford *et al*^26^ were used to assess participants’ susceptibility to tobacco smoking: ‘If one of your friends offered you a cigarette, would you smoke it?’, ‘Do you think you will smoke a cigarette at any time during the next year?’, and ‘Do you think you will be smoking cigarettes at 18 years old?’ All were rated on four-point scales: *definitely not, probably not, probably yes, definitely yes*. Participants were considered susceptible if they selected any option other than *definitely not*.

Measures taken only in the groups exposed to e-cigarette adverts.

##### Appeal of e-cigarette adverts

This was assessed by asking: ‘How much do you like the advert (not the product)?’ rated on scales from *1=Not at all* to *4=A lot*.^30^ Responses to all 12 adverts were averaged into a single index (α=0.83).

##### Interest in buying and trying e-cigarettes following adverts

This was assessed by responses to one item: ‘Does this advert make you want to buy and try this product?’ with scores ranging from *1=Not at all* to *4=Yes, a lot*.^30^ Responses were averaged across the 12 adverts (α=0.90).

#### Other measures

##### Smoking status

This was assessed using two items ‘Have you ever smoked a cigarette?’ and ‘Have you ever tried or experimented with cigarette smoking, even a few puffs?’ both answered on a binary *yes–no* scale.^29^

##### E-cigarette awareness and use

Two items assessed participants’ awareness and use of e-cigarettes (‘Before today, had you ever heard of e-cigarettes?’, and ‘Have you ever used e-cigarettes?’) both answered on a binary *yes–no* scale.

##### Demographic characteristics

The following characteristics were assessed: gender, age and ethnicity.

### Procedure

The study was conducted in schools. Ethics approval was obtained from the University of Cambridge's Psychology Research Ethics Committee. Prior passive parental consent was obtained, and the head-teachers of the schools acted *in-loco parentis* while the study was carried out. During data collection in the schools, participating children were reminded that they could withdraw from the study at any point.

The study materials were presented in paper–pencil format, with each child receiving a booklet corresponding to one of the three experimental conditions depending on randomisation. Participants in the flavoured and non-flavoured e-cigarette adverts conditions were each exposed to a series of 12 print-adverts in their booklets. To ensure that the children engaged with the adverts, after each advert, we asked the children to rate the appeal of the advert, and also their interest in buying and trying the product shown in the advert (see Measures section).

Participants were assigned to one of the three experimental versions of the experiment by the experimenters. Prior to the testing session the different versions of the booklets were arranged in a fixed sequence (ABC, ABC). On arrival, children chose any of the available seats. Booklets were then distributed in class. Care was taken to ensure that the starting position for issuing the sequence of booklets differed randomly between classes. To maximise the independence of individual responses children sitting on adjacent seats did not receive the same booklets. Experimenters made sure that children finishing earlier than other children remained seated until the rest of the children had finished. Once participants had completed their questionnaires, they were provided with verbal and written debrief about the nature of the study and took part in a workshop on tobacco smoking and e-cigarette use.

## Results

Examination of the distribution of the data revealed that responses to the primary and secondary outcome measures were not normally distributed. The subsequent analyses were therefore carried out using non-parametric statistical tests (for descriptive statistics see table 3). Participants who answered yes on either of the two smoking status questions (n=98), or who affirmed that they had ever used e-cigarettes (n=73, including 29 non-smokers who had only used e-cigarettes) were removed from subsequent analyses. Sensitivity analyses carried out on the full sample (including smokers and e-cigarette users) replicated the reported results.

**Table 3:** Attitudes (mean (SD)) towards tobacco smoking and e-cigarettes by experimental group

	Control (n=157)	Flavoured ads (n=166)	Non-flavoured ads (n=148)	Total (n=471)
Appeal of tobacco smoking	1.36 (0.58)	1.38 (0.64)	1.42 (0.62)	1.38 (0.61)
Appeal of using e-cigarettes	1.73 (0.85)	1.73 (0.89)	1.68 (0.83)	1.71 (0.86)
Perceived harm of smoking tobacco cigarettes	4.19 (0.53)	4.04 (0.71)	4.11 (0.67)	4.11 (0.64)
Appeal of e-cigarette adverts	–	2.12 (0.56)	1.94 (0.53)	2.03 (0.55)
Interest in buying e-cigarettes	–	1.75 (0.62)	1.48 (0.53)	1.63 (0.59)

### Appeal of tobacco smoking

The appeal of tobacco smoking was similarly low across the three experimental groups: Kruskal-Wallis test, χ^2^(2)=1.673, p=0.433, with a mean rank appeal score of 231.29 for the control condition, 229.89 for the flavoured e-cigarettes condition, and 246.22 for the non-flavoured condition.

### Appeal of using e-cigarettes

The appeal of using e-cigarettes was also similarly low across the three experimental groups: Kruskal-Wallis test, χ^2^(2)=0.235, p=0.889, with a mean rank appeal score of 239.04 for the control condition, 233.74 for the flavoured e-cigarettes condition, and 232.15 for the non-flavoured condition.

### Perceived harm of tobacco

The perceived harm of smoking tobacco cigarettes was similarly high across the three experimental groups: Kruskal-Wallis test, χ^2^(2)=1.955, p=0.376, with a mean rank harm score of 245.10 for the control condition, 224.61 for the flavoured e-cigarettes condition, and 237.54 for the non-flavoured condition.

Since reliability analyses of the perceived harm of smoking tobacco cigarettes showed that item reliability for the scale was low, we also analysed the single item that is most often used in the literature; ‘Smoking can harm your health’. This replicated the null effect reported above when using the composite measure: χ^2^(2)=1.222, p=0.543, with a mean rank harm score of 240.07 for the control condition, 230.11 for the flavoured e-cigarettes condition, and 236.71 for the non-flavoured condition.

### Susceptibility to tobacco smoking

Susceptibility to tobacco smoking was similar across the three groups. A series of logistic regression analyses contrasting the effects of the three experimental conditions on susceptibility to tobacco smoking yielded no significant results, all p values >0.441.

Measures taken only in the groups exposed to e-cigarette adverts.

### Appeal of e-cigarette adverts

Exposure to the flavoured e-cigarette adverts increased the appeal of e-cigarette adverts: Mann-Whitney test, U=10 056.500, Z=−2.777, p=0.005, whereby those who saw the flavoured e-cigarette adverts rated them as more appealing (mean rank=170.92) than those who saw the non-flavoured e-cigarette adverts (mean rank=142.45).

### Interest in buying and trying e-cigarettes

Exposure to the flavoured e-cigarette adverts increased interest in buying and trying e-cigarettes: Mann-Whitney test, *U*=9140.000, Z=−3.949, p<0.001, whereby those who saw the flavoured e-cigarette adverts expressed greater interest in buying and trying e-cigarettes (mean rank=176.44) than those who saw the non-flavoured e-cigarette adverts (mean rank=136.26).

## Discussion

In an experimental study, we found no evidence that exposing English children aged 11–16 years to adverts for candy-like flavoured and non-flavoured e-cigarettes increased the low appeal of smoking tobacco, the low appeal of using e-cigarettes, or low susceptibility to tobacco smoking. Nor did it reduce the high perceived harm of tobacco smoking. Flavoured e-cigarette adverts compared with non-flavoured adverts were, however, more appealing, and elicited greater interest in buying and trying e-cigarettes.

Our data provide no support for the renormalisation hypothesis, since exposure to e-cigarette adverts did not increase the appeal of tobacco smoking in this sample of children. However, our data suggest that certain types of e-cigarette advertising (eg, for candy-like flavoured e-cigarettes) may provide a gateway into tobacco smoking by increasing the appeal of e-cigarette adverts, and increasing interest in buying and trying e-cigarettes. Future studies could use the social learning theory and other relevant theories to test the proposed gateway and renormalisation hypotheses for the impact of e-cigarette exposure on tobacco use.^9^

These results provide the first evidence regarding the impact of exposure to e-cigarette adverts on the appeal of tobacco smoking. Whether the observed null effect of e-cigarette adverts on appeal also indicates the absence of an effect on tobacco smoking depends on the relationship between appeal and smoking behaviour. Appeal is an attitude, affective in origin, involving positive or negative feelings towards an object or behaviour (see Ajzen^31^ for a discussion of the relationship between attitudes and behaviour). Affect takes primacy in influencing many judgements and much behaviour (for reviews and models see refs 32 and 33). In keeping with this, the appeal of tobacco smoking predicts subsequent tobacco smoking in young people.^34^ ^35^ Replicating the current study using more vivid adverts, other measures of appeal (eg, IAT^36^), as well as tobacco cigarette use assessed prospectively, will increase the confidence attached to the current findings.

While we found no evidence that exposure to adverts increased the appeal of tobacco smoking, exposure to candy-like flavoured as opposed to non-flavoured e-cigarettes did increase the appeal of e-cigarette adverts and interest in buying and trying these products. This fits with the extant literature on flavoured nicotine products and their appeal to children.^16^ ^20^ ^22^ The plethora of different flavoured e-cigarettes (documented as 7764 in 2014, and rising,^15^) may explain the increases in e-cigarette use documented in the UK, the USA and Canada.^6^ ^7^ ^37^ Our findings, combined with recent prevalence figures of e-cigarette use, highlight the need for more research into the effects of flavoured e-cigarettes on young non-smokers, and how this may impact uptake of e-cigarettes, and the potential for eventual migration to tobacco smoking (gateway hypothesis).

While adverts did not affect the appeal of using e-cigarettes, they affected children's interest in buying and trying e-cigarettes. There are two main possible reasons for such a difference in effects. First, one of the outcome measures taps into more general appeal of long-term use of e-cigarettes, whereas the other measure taps into shorter term interest in buying and trying e-cigarettes without necessarily making a commitment for long-term use. Second, appeal of using e-cigarettes was measured in general terms, whereas interest in buying and trying e-cigarettes was measured specifically with reference to the adverts participants were exposed to. Future studies should further examine these differential effects.

### Strengths, limitations and suggestions for future work

Our study is the first, to the best of our knowledge, to use an experimental design to examine the impact of exposing children to adverts for flavoured and non-flavoured e-cigarettes.

One limitation pertains to the primary endpoint, which was a measure of attitude and not behaviour. Future studies would benefit from measuring actual smoking behaviour. Similarly, tobacco smoking carries a stigma, therefore, it is possible that children gave socially desirable answers regarding the appeal of tobacco smoking. Future studies would benefit from using implicit measures to examine the appeal of tobacco smoking. Since interest in buying and trying e-cigarettes was examined after each advert, it was not possible to ask children for their interest in buying and trying tobacco cigarettes, since adverts of tobacco were not shown in this study. Future research could consider a condition where tobacco adverts are shown, in order to gauge parallel responses to all the measures for both e-cigarette and tobacco products.

Furthermore, children were exposed to still images of adverts. Future studies should examine the effects of e-cigarette adverts using different formats such as videos. Examining children's exposure to e-cigarettes via their peers in their naturalistic environments would also complement the current findings. In addition, we only had one control condition in which children were not exposed to any adverts. Future studies could extend the design to incorporate a control condition similar to those in the e-cigarette advert conditions (eg, adverts for neutral products, like stationery). This would ensure that task demands are kept equal across all conditions.

While we found the same pattern of results when carrying out sensitivity analyses on the full randomised sample including tobacco smokers and e-cigarette users, it is possible that our results underestimate the effects of e-cigarette adverts on tobacco smoking, since by removing the smokers and e-cigarette users we may have ended up with a sample of the least susceptible children. Future studies should extend the present findings by specifically sampling children who are tobacco smokers and e-cigarette users.

### Implications for policy

Currently, across Europe and the USA, marketing and advertisement of e-cigarettes is unregulated. In the UK, before EU-wide regulation takes place in 2016, the Committee on Advertising Practice has issued rules for the advertising of e-cigarettes.^12^ Key aspect of these rules is that e-cigarette adverts must not be likely to appeal to people under 18 years, to non-smokers or non-nicotine users, and must not have models appearing younger than 25 years. These interim rules do not provide any explicit prohibitions regarding the advertising of candy-like flavours.

Our results point to a need for further examination of the rules surrounding e-cigarette advertising especially in light of the growing popularity of e-cigarettes among children.^6^ ^7^ ^37^ While our study suggests that e-cigarette adverts do not increase the appeal of tobacco smoking, allowing us to be cautiously optimistic that e-cigarette advertising does not directly renormalise tobacco smoking, our results provide evidence that children find adverts for candy-like flavoured e-cigarettes more appealing than adverts for non-candy-like e-cigarettes, potentially serving as a gateway into tobacco smoking. In addition to the gateway concerns, the heightened appeal of the adverts and interest in buying e-cigarettes in children arising from adverts promoting candy-like flavoured e-cigarettes is of concern in and of itself in view of the dangers to the developing brain arising from nicotine exposure and addiction,^8^ ^17^ ^38^ as well as the unknown long-term physiological effects of using e-cigarettes and secondhand exposure to vaping.^39^

**What this paper adds**

- E-cigarette use is rising among children and adolescents, with fears that their use could lead to tobacco smoking.
- Internal tobacco industry documents show that young people find nicotine products with candy-like flavours more appealing than those without.
- E-cigarettes are currently marketed in over 7764 different flavours.
- There are currently no studies examining the impact of e-cigarette adverts, with or without flavours, on the appeal of tobacco smoking in children.
- Adverts promoting candy-like flavoured or non-flavoured e-cigarettes did not increase the current low appeal of tobacco smoking.
- Adverts promoting candy-like flavoured compared with non-flavoured e-cigarettes were more appealing and elicited greater interest in buying and trying e-cigarettes among English children aged 11–16 years.
- Further studies replicating and extending the current research are needed to elucidate the impact of candy-like flavoured and non-flavoured e-cigarette adverts.
